# Neuromelanin? T_1_ shortening is a necessary condition

**DOI:** 10.1093/braincomms/fcag214

**Published:** 2026-06-09

**Authors:** Takashi Watanabe

**Affiliations:** Medical Scanning Musashikosugi Clinic, Jiaikai Healthcare Corporation, Yokohama, Kanagawa Prefecture 221-0835, Japan

I wish to provide a constructive critique of the recent paper^1^ by Pérot *et al*. in *Brain Communications.* My observations are intended to engage in scholarly correspondence and further the understanding of this important topic. In general, the longitudinal relaxation time (T_1_) and transverse relaxation time (T_2_) of the white matter (WM) are shorter than those of the grey matter (GM). The longitudinal and transverse relaxation rates (R_1_ and R_2_), the reciprocals of T_1_ and T_2_, respectively, of WM are higher than those of GM. These relationships result from lower water content in WM than in GM. Accordingly, WM appears brighter than GM in R_1_ map, R_2_ map, R_2_* map, magnetization transfer (MT) ratio map and T_1_-weighted MRI, while GM appears brighter than WM in T_1_ map, MT-MRI and proton-density-weighted MRI. Now, so-called ‘neuromelanin (NM)-sensitive MRI (NM-MRI)’ is technically ‘MRI with intense on- and off-resonance excitation’ or ‘T_1_-weighted MRI with MT.’ First of all, as in standard T_1_-weighted MRI, repeated on-resonance excitation saturates free water protons, e.g. in the cerebrospinal fluid. Additionally, off-resonance excitation saturates (through MT) bound water protons in proportion to the degree of T_1_ shortening (Fig. 1B in ref.^2^). For example, macromolecules in the myelin shorten the T_1_ of bound water protons and thus ‘increase’ the signal intensity of WM most effectively, while MT ‘decreases’ the signal intensity of WM most effectively. Consequently, image contrast derived from T_1_ difference in brain is offset by MT, so that image contrast derived from proton density remains. In other words, MT turns T_1_-weighted MRI of the brain into proton-density weighted.^2^ Thus, as shown in Fig. 3A of the paper by Pérot *et al*.,^1^ the cerebral cortex and the principal cell layers in the hippocampal formation appear brighter than WM in agreement with a previous work.^3^ In a recent correspondence on NM-MRI,^2,4^ both sides are in agreement that the hyperintensities are present in the cerebral cortex and striatum as well as in the substantia nigra pars compacta (SNpc) and locus ceruleus (LC) in human, regardless of NM content (Fig. 1A in ref.^2^). This observation indicates that NM-MRI is neither specific nor sensitive to NM but is sensitive to another factor. Further, both sides are in agreement that SNpc/LC exhibit higher proton density and lower macromolecular-to-free-water pool size ratio. In fact, NM-MRI signal intensity positively correlates with T_1_ as well as with the water content:^2^ T_1_ values of the hyperintense structures (i.e. the cerebral cortex, striatum, SNpc and LC) are ≥0.99 s, while T_1_ values of the hypointense structures (e.g. the thalamus, globus pallidus and WM) are ≤0.95 s (Fig. 1A, Fig. 1B and Table 1 in Watanabe^2^); the water contents of the cerebral cortex, striatum and SN are ≥0.765, while the water contents of the thalamus, globus pallidus and WM are ≤0.734. These indicate that NM-MRI signal intensity depends on the proton-density effect of water. In other correspondence,^5^ both sides are in agreement that the NM-MRI signal cannot be ascribed to NM but to the water content. On this assumption, compositional changes in brain tissue (e.g. SNpc) may be estimated from T_1_ and NM-MRI signal intensity (Table 1 in ref.^6^). It is true that NM-MRI is not only sensitive to proton density but also to paramagnetic ions,^3^ even if only at unphysiologically high concentration (** in Table 1 of ref.^6^). Notably, it is rather paramagnetic ions than NM that NM-MRI is sensitive to. According to Trujillo *et al*.,^7^ metal-free NM has a negligible effect on MT. Only the presence of iron results in a decreased MT. The term ‘NM sensitive’ is thus misleading because it gives readers a false impression that this MRI is particularly sensitive to NM. Accordingly, T_1_ shortening by NM *in vitro*, which only occurs at such high concentration as unlikely *in vivo,* does not cause MT reduction.^8^ SN/LC hyperintensities are thus not ascribed to NM but to ‘a lower macromolecular fraction.’

The present paper by Pérot *et al*.^1^ shows representative images of R_1_, MT ratio, R_2_* and R_2_ of animal brain as Fig. 4A, Fig. 4B, Fig. 5B and Fig. 5C, respectively. Here, WM appears brighter than GM in MT ratio map, R_2_* map and R_2_ map. In R_1_ map (Fig. 4A), however, WM appears darker than GM. This image contrast is exactly the reversal of the standard *R*_1_ map, i.e. is the same as the standard T_1_ map. Thus, firstly, I wish to ask how Fig. 4A is generated because it is this R_1_ map that the conclusion of this study entirely relies upon. With regard to Supplementary material, I assume that such a map as Fig. 4A was not used for evaluation (e.g. in Fig. 4D) but that a T_1_ map may be simply carelessly mistaken for an R_1_ map only to be shown as Fig. 4A. In any case, it is of note that such NM-MRI signal increase induced by T_1_ shortening was observed so far only with the use of strongly T_1_-shortening paramagnetic ions like manganese^3^ (Mn^2+^) or gadolinium^9^ (Gd^3+^). Secondly, as given in the subsection ‘Clinical MRI protocol,’ ‘normalized signal intensity (NSI) of the SN was computed in comparison with a background region including the tegmentum and superior cerebral peduncle.’ Then, the initial NSI increase is ascribed to NM accumulation, whereas the following NSI decrease is ascribed to neurodegeneration. Here, however, T_1_ is not measured. Thus, I wish to ask whether e.g. an initial signal decrease in the background region (e.g. due to water content decrease) appears as the initial NSI increase in SNpc.

As shown in the present animal study,^1^ a signal increase in MRI is attributable to a specific molecule, e.g. NM, only when T_1_ of the tissue with the molecule is shortened compared to T_1_ of tissue without the molecule. In other words, demonstration of T_1_ shortening of NM-containing tissue (e.g. SNpc after injection in the animals of the present study,^1^ both SNpc and LC in human) is a necessary condition for ascribing the signal increase to NM. It is not a sufficient condition: T_1_-shortening effect of other molecules (e.g. paramagnetic ions that are not associated with NM) cannot be excluded. Conversely, a signal increase is not attributable to a specific molecule (except water) when T_1_ of the tissue with the molecule is prolonged compared to T_1_ of tissue without the molecule. In other words, demonstration of T_1_ prolongation of NM-containing tissue, e.g. LC, is a sufficient condition for ascribing the signal increase to proton density:^10^ Except water there is no molecule that prolongs T_1_. To summarize, as evidence of T_1_-shortening effect of NM in human, I wish to see (i) T_1_-shortening effect of NM *in vitro* at concentration as *in vivo*, (ii) T_1_ shortening of both SNpc and LC compared to other GM in human and (iii) T_1_ shortening of SNpc which spatiotemporally corresponds to the initial NSI increase in human.

## Competing interests

The author reports no competing interests.

## Data Availability

Data sharing is not applicable to this article since no new data were generated or analysed.
